# Computational analyses for cancer biology based on exhaustive experimental backgrounds

**DOI:** 10.20517/cdr.2019.33

**Published:** 2019-09-19

**Authors:** Jun Koseki, Masamitsu Konno, Hideshi Ishii

**Affiliations:** ^1^Department of Medical Data Science, Osaka University, Osaka, 565-0871, Japan.; ^2^Department of Frontier Science for Cancer and Chemotherapy, Osaka University, Osaka, 565-0871, Japan.

**Keywords:** In silico drug design, trans-omics analysis, computational analyses, cancer biology

## Abstract

Antitumor drug therapy plays a very important role in cancer treatment. However, resistance to chemotherapy is a serious issue. Many studies have been conducted to understand and verify the cause of chemoresistance from multiple points of view such as oncogenes, tumor suppressor genes, DNA mutations and repairs, autophagy, cancer stemness, and mitochondrial metabolism and alteration. Nowadays, not only medical data from hospitals but also public big data exist on internet websites. Consequently, the importance of computational science has vastly increased in biological and medical sciences. Using statistical or mathematical analyses of these medical data with conventional experiments, many researchers have recently shown that there is a strong relationship between the biological metabolism and chemoresistance for cancer therapy. For example, folate metabolism that mediates one-carbon metabolism and polyamine metabolism have garnered attention regarding their association with cancer. It has been suggested that these metabolisms may be involved in causing resistance to chemotherapy.

## Introduction

Incidence of cancer is increasing worldwide, and there are some challenges in cancer treatment, including resistance to antitumor drugs. The drug resistance in cancer treatment can be of two types: acquired and congenital. Cancer therapy is typically very effective at the start of the treatment, with tumor degeneration being observed; however, recurrence and metastasis of cancer are often observed thereafter. With chemotherapy, cancer progression is a grave problem. Therefore, several studies have investigated the causes of chemotherapy resistance from multiple points of view such as oncogenes, tumor suppressor genes, DNA mutations and repairs, autophagy, cancer stemness, and mitochondrial metabolism and alteration^[1-7]^. Although each one of these individually is not a cause of drug resistance, but the complex relationship among them may result in chemoresistance. Because of this relationship, it has been recently determined that differences in the biological metabolism pathways regarding the presence or absence of the characteristic of chemoresistance are related to drug resistance in cancer therapy. In this review, we aim to summarize the research on antitumor drug resistance and cancer cell characteristics.

## Oncogenes and tumor suppressor genes

Cancer tissue is considered to be a population of abnormal cells that arise from normal cells. Normal cells proliferate depending on the surrounding conditions, whereas cancer cells continue to uncontrollably proliferate. Cancer occurs as a result of errors in the genes of normal cells over many years^[8-12]^. There are two types of gene errors: mutations and epigenetic changes. These errors occur in cases where gene activation causes cell proliferation and acts as an accelerator (activation of oncogenes) and in cases where a braking action for arresting cell growth is not activated (inactivation of tumor suppressor genes). Therefore, many studies have reported on oncogenes and tumor suppressor genes.

Typical examples of oncogenes include *c-MYC*^[13-15]^ and *RAS*^[16-18]^. *MYC* family of genes is known to cause cancer by an overexpression of the product protein, c-MYC, for some reason combined with a loss of the original function of cell cycle control. Translocation of the *c-MYC* gene close to a transcriptionally active immunoglobulin gene results in overexpression. This behavior has been observed in human Burkitt’s lymphoma, diffuse large B-cell lymphoma, and some carcinomas. However, in some cancers such as colon cancer, there is an exception where the survival rate may be better if the expression is high^[19,20]^. The *RAS* family of genes produces a RAS protein that has an important role in transmitting signals that promote cell proliferation. There are three types of *RAS* gene: *KRAS*, *NRAS*, and *HRAS*. It is well known that the epidermal growth factor receptor (EGFR) on the cell surface is involved in one of the mechanisms involved in the proliferation of cancer cells. Normally, the *RAS* gene controls cell growth. However, in case of a *RAS* gene with a significant mutation, cell proliferation signals continue to be emitted, even if EGF and EGFR are not bound. As a result, cancer cell proliferation is activated.

Many studies have reported on tumor suppressor genes. The *P53* gene^[21-24]^ and retinoblastoma1 (*RB1*) gene susceptibility^[25,26]^ are the representative examples. They are known to play important roles in cell death induction, cell proliferation suppression, and DNA repair. P53 is a stress-induced transcription factor that can promote cell cycle arrest, apoptosis, and senescence. In addition, it is involved in the regulation of metabolic pathways and cytokines required for embryo transfer. Because P53 plays these important roles, it would be natural that any mutation in the *P53* gene will promotes P53 protein dysfunction and cancer development. Furthermore, *RB1* is a known tumor suppressor gene, which is characterized as a “gatekeeper.” Mutations in the *RB1* gene occur in almost all familial and sporadic forms of RB1. The *RB1* gene produces the RB1 protein, which plays a role in the repression of the E2F transcription factor family. For tumor initiation, this inactivation is a rate-limiting step. Thus, the relationship between various genes (oncogenes and tumor suppressor genes) and cancer has been reported, and it is a well-established fact that cancer results from genetic errors. Any change in the function of the above genes induces epigenetic changes and biological metabolisms in cancer cells.

## Metabolism in normal cells and cancer cells

There are significant differences in metabolism between normal cells and cancer cells^[27]^. The Warburg effect is representative of the metabolism in cancer cells; however, our understanding of the effect in terms of cancer biology remains incomplete, as is true for other metabolic changes that characterize cancer tissue^[28-31]^. In normal cells, glucose is metabolized to carbon dioxide in the presence of oxygen, although it produces large amounts of lactic acid only in hypoxic conditions. However, in cancer cells, a large amount of lactic acid is produced regardless of the amount of oxygen. Glycolysis in cancer cells is closely related to the rate of adenosine 5ʹ-triphosphate (ATP) consumption^[32]^. Cancer cells need to balance the catabolism that produces ATP with the need for metabolism that promotes biosynthesis and cell division throughout the cell. The aerobic glycolytic system can reportedly balance many of the metabolic requirements for the growth of cancer cells. We have mentioned above that a large amount of lactic acid is produced in cancer cells, and it has become clear that lactic acid is used as a nutrient in some cancer cells^[33]^.

In many cancer cells, metabolic pathways are maintained by oxidative phosphate using multiple nutrients. Glucose is not the only substrate used for oxidative phosphorylation in cancer cells^[34,35]^. A pathway that metabolizes glutamine to α-ketoglutarate has been reported^[36]^. Glutamine is required for cell growth in many cancer cells, and it is more abundantly metabolized than other non-essential amino acids^[37,38]^. Glutamine metabolism is a known source for the synthesis of macromolecules such as nucleotides, proteins, and lipids. However, it also supports nicotinamide adenine dinucleotide phosphate production and anaplasia in the proliferation of cancer cells. Thus, some amino acids and ions are also important factors associated with survival and maintenance of cancer cells.

In addition, the relationship between oxidative damage and cancer malignancy has also been reported for a long time^[39,40]^. Reactive oxygen species (ROS), such as hydrogen peroxide, superoxide, hydroxyl radical, and singlet oxygen, is well known. It has an important role in immune function and defense against infection. It is also used as a physiologically active substance for signal transduction, cell differentiation, and apoptosis. However, high level of ROS is very toxic to cells. It is reported that the expression level of antioxidant enzymes in cancer is changed both *in vivo* and *in vitro* experiments^[39]^. Generally, oxidative stress and redox signaling are involved in the development of cancer and ROS could influence the phenotypic behavior of cancer cells and their responsiveness to therapeutic interventions^[40]^. On the other hand, it has also been reported that the enhancement of oxidative stress in normal cells limits tumor development and tumor progression. And then, if the oxidative stress is not controlled, the level of ROS turns higher to the point of causing senescence or apoptosis and turns into a tumor suppressor^[41]^. Then, the fact means that strategic targeting of antioxidant system could be effective for undermining new tumor cells^[42]^.

Further, it was reported that Raf/MEK/ERK pathway had an important role to drug resistance^[43]^. This pathway is a signal transduction system responsible for cell growth, and the abnormal activation of this pathway is known that normal cells turn into cancer cells. In some type of cancer, this pathway regulates the expression level of drug pumps and anti-apoptotic molecules. McCubrey *et al*.^[43]^ show that ectopic expression of Raf induces increased expression of Mdr-1 and Bcl-2 associated with drug resistance.

**Figure 1:**
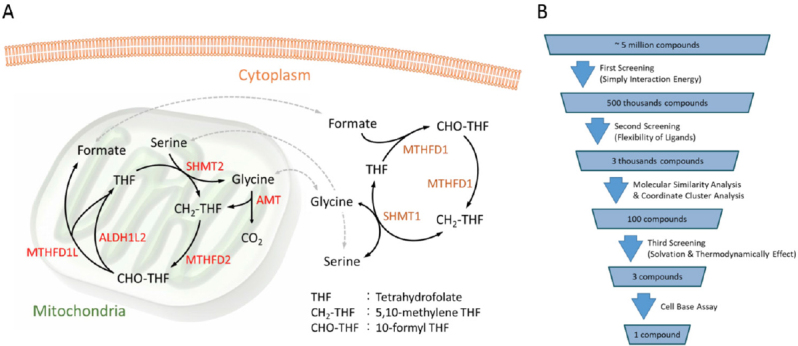
A: Schematic diagram of the metabolic cycle of THF and the one-carbon (1C) metabolic pathway in folate metabolism, which are regulated by SHMT2, AMT, MTHFD2, ALDH1L2, and MTHFD1L in mitochondria and that of MTHFD1 and SHMT1 in cytoplasm; B: Candidate compound search procedure with in silico molecular docking and a cell-based assay. THF: tetrahydrofolate; SHMT2: serine hydroxymethyltransferase 2; AMT: aminomethyltransferase; MTHFD2: methylenetetrahydrofolate dehydrogenase 2; ALDH1L2: aldehyde dehydrogenase 1 family member L2; MTHFD1L: methylenetetrahydrofolate dehydrogenase 1 like; MTHFD1: methylenetetrahydrofolate dehydrogenase 1

Recently, the significance of the mitochondrial metabolism is also garnering attention. In some cancers, mutations have been observed in mitochondrial enzyme genes; however, the mitochondrial function is not lost in many cancer cells^[44]^. Folate metabolism in mitochondria is known to mediate one-carbon metabolism^[45-52]^ [Figure 1A]. It is closely related to cancer cell characteristics^[53,54]^. Serine hydroxymethyltransferase 2, methylenetetrahydrofolate dehydrogenase 2 (*MTHFD2*), and aldehyde dehydrogenase 1 family member L2 are known as the genes that control the metabolic cycle of tetrahydrofolate in mitochondria. In fact, among patients with colorectal cancer and lung adenocarcinoma, those with high expression of these genes have a shorter overall survival rate than those with low expression^[53]^.

As mentioned above, the cancellation of normal cells greatly alters the biological metabolism of cancer cells compared with that of normal cells. It is caused by genomic or epigenetic errors that are important for carcinogenesis. Generally, many cancer driver genes enhance nutrient intake. This means that the transformation of cancer cells is strongly related to increased glucose uptake^[55,56]^. In human cancers, activation of the phosphatidylinositol-3 kinase (PI3K) signaling pathway is frequently observed. Reportedly, PI3K is involved in glucose metabolism through the insulin signal leading to glucose uptake^[57]^. Conversely, just as some gene mutations lead to oncogenesis, metabolic errors in some cells may also be a direct cause of oncogenesis. These facts indicate the possibility of treating cancer by controlling certain metabolisms. Therefore, drug discovery research has also been performed regarding antitumor drugs targeting certain metabolisms^[54]^. Our group has performed *in silico* molecular docking to search for effective candidate compounds [Figure 1B]. Consequently, very promising compounds potentially targeting MTHFD2 could be found earlier. Our statistical analyses using clinical data led us to predict that compared with the conventional target dihydrofolate reductase, MTHFD2 is a more efficient drug target for cancer therapy with less side effects in normal cells^[53]^.

So far, we have focused on the differences between normal cells and cancer cells. However, cancer tissue is not a group of cancer cells that have homogeneous properties but a collection of heterogeneous cancer cells that exhibit different biological characteristics. A cancer stem cell model has been proposed to explain this heterogeneity^[8,9]^.

## Cancer stem cell model

Reportedly, tumor cells exhibit heterogeneity in their proliferative ability. The fact that only some cells have the ability to form tumors was mentioned for the first time in a paper on lymphoma in mice^[58]^. Transplantation of human hematopoietic stem cells into NOD/SCID mice has shown to repopulate human hematopoietic cell lines over time^[59]^, and when similar transplantation is performed using hematopoietic stem cells of patients with leukemia, mice develop leukemia^[60]^. These behaviors indicate that leukemia develops when hematopoietic stem cells become tumor-initiating cells. The tumor-initiating cells are defined as cancer stem cells (CSCs) that have three stem-cell-like characteristics: differentiation, self-renewal, and proliferation maintenance. CSCs have been defined in many solid tumors such as breast cancer^[61]^, glioblastoma^[62]^, head and neck cancer^[63]^, some digestive organ cancers^[64,65]^, and other types of cancer. CSCs have the property of therapy resistance^[3,7]^. Thus, many biomarkers have been reported to define and distinguish these CSCs^[66,67]^. Recently, it has been reported that CSCs in breast cancer and gliomas may be identified using fluorescent protein with a degron motif, which is broken down by proteasomes. Because the proteasome activity in CSCs is lower than that in non-CSCs, CSCs yield fluorescence and non-CSCs do not^[68]^. Furthermore, it has been verified in other types of cancers that the cells identified using the abovementioned techniques retain cancer stem-cell-like properties and maintain chemoradiation resistance^[69,70]^.

Epithelial-mesenchymal transition (EMT) is a change because of which epithelial cells lose their characteristics obtained at differentiation and acquire mesenchymal traits. It has been reported that EMT contributes to tumor progression by enhancing the infiltrative metastatic potential of cancer cells^[71,72]^ and by increasing the resistance to antitumor drugs^[73]^.

CSCs have very different biological characteristics from non-CSCs constructing majority of the tumor tissue. CSCs exhibit characteristics such as very slow cell cycle and drug resistance. Therefore, for complete cure of cancer, it is necessary to completely kill CSCs. Some recent studies have shown the possibility that epigenetic regulation can control cancer stemness^[74,75]^. Histone demethylating enzyme plays a role in the regulation of cell proliferation and maintenance of stemness. Therefore, a reduced activity of this enzyme leads to cancer cell death. A study has focused on targeting histone demethylase using small molecules^[76]^; however, there are many challenges to cancer therapy targeting CSCs.

A little aside from this, it is essential for cancer cell growth to have unlimited replication capacity. On the other hand, in normal cells, the number of cell divisions is limited. It is known that the unlimited proliferation ability of cancer cells is involved in telomeres protecting the ends of chromosomes^[77-80]^. In normal cells, their length decreases as cell division repeats, and eventually their ability to protect the ends of chromosomes is lost. Telomerase is a DNA polymerase that adds a telomeric repeat to the end of telomeric DNA. Telomerase is almost absent in normal cells but is expressed at significant levels in most cancer cells as is the case with normally stem cells. Telomere activity is associated with resistance to both replicative senescence and induction of crisis apoptosis. On the other hand, it is reported that suppression of telomere activity leads to activation of either replicative senescence or crisis apoptosis.

## Computational analyses

Advances in observational techniques for experimental equipment have also increased the volume of the available data. Consequently, omics analyses such as those of methylome, transcriptome, proteome, and metabolome have enabled more comprehensive understanding of the biological molecules for exhaustive research. As the performance of computing devices has improved, rapid advances have made it possible to collectively calculate large volumes of data. With advances in the computer technology, innovative analytical methods have been developed to evaluate the correlations between multiple experimental data. These new analytical methods also make it possible to gain new knowledge that could not be achieved with conventional methods. To date, many studies using computational science have been conducted in the field of cancer research^[81,82]^.

**Figure 2:**
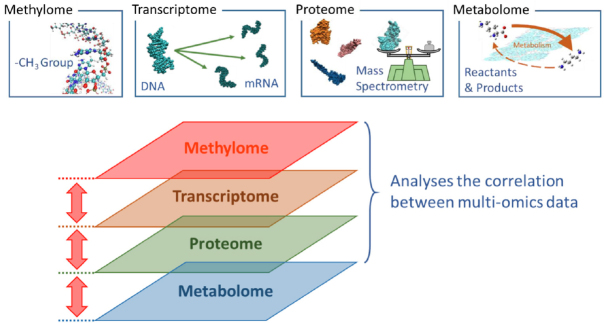
Schematic diagram of trans-omics analysis. There is a correlation between each omics data (layer), and some changes should affect the data of the separate upper and lower layers. New findings are derived by a comprehensive analysis of each layer and a correlation analysis between the layers

As mentioned above, the amount of biological data in the medical research field is large. Therefore, conventional statistical methods have been used as a very powerful tool. Recently, trans-omics methods were proposed, which is for mechanically reconstructing a large-scale network across multiple omics layers with integration multi-omics data from samples prepared under the same conditions^[83,84]^. However, it was said that some technical and analytical improvements will still be required to make this approach a reliable approach^[83]^. On the other hand, one of the trans-omics method developed by by Koseki*et al*.^[85]^ and Konno *et al*.^[86]^ has been groundbreaking in identifying some biological metabolisms that may be involved in anticancer drug resistance in cancer cells. Trans-omics analyses are a method of grasping the global network of biological molecules by hierarchically linking multiple omics data using statistics or mathematical techniques [Figure 2]. The experimental results obtained using a conventional single omics analysis are just a snapshot at one time. However, the trans-omics method, using some multi-omics data, has made it possible to predict (interpolation/extrapolation) changes over time through correlation analyses among the data. Koseki *et al*.^[85]^ have created a novel trans-omics method to predict the changes in a specific reaction rate and performed prediction analyses using transcriptome and metabolome data to compare CSCs and non-CSCs. Furthermore, they have suggested that the polyamine metabolic pathway plays an important role in antitumor drug resistance in CSCs. The polyamine metabolism involves the reaction from ornithine to spermine via putrescine and spermidine. In non-CSCs, the inflow to putrescine stands out after exposure to an antitumor agent, and its reaction rate slows over time. Conversely, it appears that the polyamine reaction in CSCs is controlled in a manner that increases ornithine to a certain level. Furthermore, high-level polyamines inhibit lysine demethylase 1A (LSD1) enzyme activity because polyamine oxidase (a polyamine catabolic enzyme) is highly similar in overall structure to LSD1^[87]^. Thus, it has been proposed that it could result in the death of cancer cells. This means that CSCs regulate polyamine metabolism to protect their own survival from antitumor agents. Application of computational analyses in this manner has made it possible to identify biological reactions that contribute to antitumor drug resistance.

## Conclusion

Earlier, most computational analyses were used in physical and chemical sciences rather than in life and medical sciences. However, they are now widely used in many scientific areas. The computational approach is not only complementary to the results of conventional experimental research but it can also significantly improve the speed and accuracy of analysis. Furthermore, using computational analyses makes it possible for one to achieve new results and interpretations that may not be understood using only conventional experimental systems. Through computational analyses of differences in cancer cell characteristics observed in some experiments such as those on methylomes, transcriptomes, and metabolomes, it has become possible for researchers to extract the biological metabolism that influences cancer cell characteristics such as antitumor drug resistance. Therefore, computational analyses such as statistical and trans-omics analyses may become very powerful tools in answering some scientific interrogations in the life and medical sciences.

## Declarations

### Copyright

© The Author(s) 2019.
